# Non-invasive remote limb ischemic postconditioning protects rats against focal cerebral ischemia by upregulating STAT3 and reducing apoptosis

**DOI:** 10.3892/ijmm.2014.1873

**Published:** 2014-07-31

**Authors:** ZHIGANG CHENG, LING LI, XUEYING MO, LU ZHANG, YONGQIU XIE, QULIAN GUO, YUNJIAO WANG

**Affiliations:** Department of Anesthesiology, Xiang Ya Hospital, Central South University, Changsha, Hunan 410008, P.R. China

**Keywords:** ischemic postconditioning, cerebral ischemia, apoptosis, inflammation

## Abstract

The signal transducer and activator of transcription 3 (STAT3) signaling pathway has been implicated in cell apoptosis and inflammatory processes. Ischemic preconditioning (IPC) and ischemic postconditioning (IPTC) inhibit both of these processes. In the present study, we investigated the role of phosphorylated STAT3 (p-STAT3)-mediated apoptosis and inflammation following non-invasive remote limb IPTC (NRIPoC) using a classic rat model of focal cerebral ischemia. Forty-five adult male Sprague-Dawley rats were divided randomly into 3 groups (n=15 per group): the sham-operated, ischemia/reperfusion (I/R) and NRIPoC groups. NRIPoC was implemented at the beginning of reperfusion. At 24 h after cerebral reperfusion, we evaluated the neurological deficit score (NDS), assessed the cerebral infarct size and tissue morphology, and evaluated neuronal apoptosis. The protein expression levels of Bcl-2, Bax, nuclear factor-κB (NF-κB), tumor necrosis factor-α (TNF-α) and p-STAT3 in the penumbra region were assessed by western blot analysis. The cerebral infarct volume, the number of apoptotic cells and the protein expression levels of Bcl-2, Bax, NF-κB and TNF-α were all found to be increased in the I/R group compared with the sham-operated group. However, these levels were decreased in the NRIPoC group compared with the I/R group. The number of apoptotic cells in the penumbra in the I/R group was increased compared with that in the NRIPoC and sham-operated groups. The protein expression of p-STAT3 was increased in the NRIPoC group compared with the sham-operated and I/R groups. These results indicate that the protective effects of NRIPoC against cerebral I/R injury may be related to the attenuation of neuronal apoptosis and inflammation through the activation of STAT3.

## Introduction

Ischemic stroke is a major cause of death and disability worldwide, and the clinical prognosis of acute cerebral ischemia is poor (1,2). The early reperfusion after cerebral ischemia is essential for the viability and functional recovery of the brain; however, the arrival of blood oxygen to the ischemic tissue causes ischemia/reperfusion injury (IRI), which induces further damage (3). Since 1986, when Murray *et al* (4) first described a classic phenomenon they termed ischemic preconditioning (IPC), it has been repeatedly confirmed in animal models that IPC and ischemic postconditioning (IPTC) are powerful endogenous protective strategies against IRI of multiple organs, including the heart, brain and kidneys (8). However, the clinical implementation of IPC and IPTC as a means of protection against IRI is not desirable or feasible in most clinical circumstances due to the unpredictability of organ ischemia and the possibility that added ischemia may result in dangerous complications (9). The application of brief episodes of ischemia to a tissue remote from the primary ischemic organ (e.g., heart or brain) using the techniques of remote ischemic preconditioning (RIP) or remote ischemic postconditioning (RIPoC) may confer cerebral/cardiac protection, and RIPoC can overcome the aforementioned issues previously associated with IPC and IPTC (10–17). Both RIP and RIPoC have been recognized as applicable strategies for the protection against cerebral and myocardial IRI (11,13,18–22). A further refinement of RIPoC, non-invasive remote ischemic postconditioning (NRIPoC), can be applied in a wider range of clinical settings for cerebral and myocardial IRI due to its practicality as a non-invasive technique. NRIPoC is a relatively recent innovation (11,23–25), and its fundamental biology is not yet well understood.

Apoptosis and the inflammatory response both play fundamental pathogenic roles in cerebral IRI (26–32). Tumor necrosis factor-α (TNF-α) is the most important pro-inflammatory cytokine involved in cerebral IRI, and its effects are observed throughout the development of inflammation (33–35). Nuclear factor-κB (NF-κB) regulates the gene expression of inflammatory factors involved in the ischemia/reperfusion (I/R) of the cerebral tissue (36,37). The inflammatory response can induce apoptosis by regulating apoptotic signals during cerebral IRI (38–41). Evidence indicates that the Janus kinase (JAK)/signal transducer and activator of transcription 3 (STAT3) signaling pathway transduces stress-activating extracellular chemical signals into cellular responses for a number of pathophysiological processes, such as immunity, inflammation and apoptosis, and is involved in cerebral IRI (42–45). We hypothesized that the NRIPoC-induced neuroprotective effects may be associated with the activation of the STAT3 signaling pathway, the inhibition of the inflammatory response and the regulation of apoptosis. To explore this hypothesis, we performed experiments to evaluate the roles of apoptosis, the inflammatory response and STAT3 signaling in cerebral protection conferred by NRIPoC during focal cerebral IRI in an *in vivo* rat model.

## Materials and methods

### Animals

Male Sprague-Dawley rats weighting 250–300 g were purchased from the Center of Laboratory Animals of Central South University, Changsha, China. The rats were placed in a room with a controlled environment with a 12-/12-h light/dark cycle and allowed access *ad libitum* to standard rodent chow and tap water. This study was approved by the Institutional Animals Ethics Committee of Central South University and was conducted in accordance with the Guidelines for the Care and Use of Laboratory Animals provided by the National Institutes of Health (NIH publication no. 80–82).

### Model of focal cerebral ischemia

Middle cerebral artery (MCA) occlusion (MCAO) was carried out as previously described (46). The animals were anesthetized by an intraperitoneal injection of 300 mg/kg chloral hydrate. Heating lamps were used to maintain rectal temperature at 37–37.5°C. The right common carotid artery (CCA), external carotid artery (ECA) and internal carotid artery (ICA) were exposed through a midline neck incision, and the ECA was ligated close to its origin with a 3-0 silk suture. A 0.26-mm monofilament nylon suture with a blunt tip (Beijing Shandong Industrial Corp., Beijing, China) was inserted into the ICA, and advanced 18–20 mm until mild resistance was felt, effectively occluding the MCA. After 90 min of MCAO, the monofilament nylon suture was removed and ICA perfusion was restored.

### Experimental protocol

**Figure 1:**
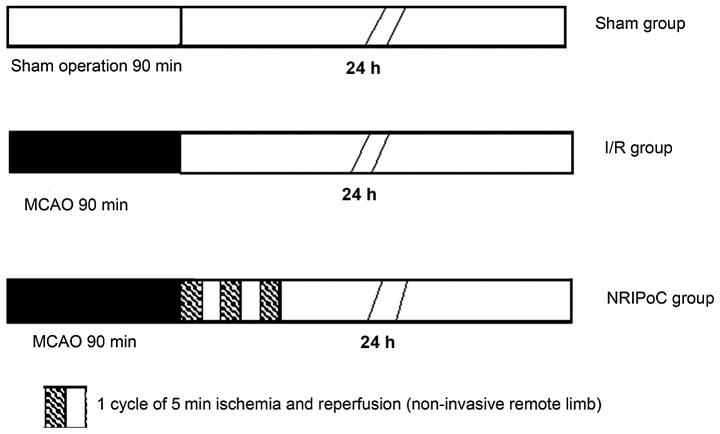
Illustration of the experimental protocol. The sham-operated (sham) group underwent the operation without right middle cerebral artery (MCA) occlusion. Both the ischemia/reperfusion (I/R) and non-invasive remote ischemic postconditioning (NRIPoC) groups underwent MCA occlusion (MCAO) by occlusion of the right MCA for 90 min, followed by 24 h of reperfusion. In addition, the NRIPoC group underwent sequential I/R of the right hind limb, using 3 cycles of 5 min of ischemia and 5 min of reperfusion following MCA reperfusion. Five rats were randomly selected from each group to assess the cerebral infarction volume [histological and 2,3,5-triphenyltetrazolium chloride (TTC) staining] and for western blot analyses.

In total, 45 rats were randomly assigned to 3 groups (n=15 in each group): the sham-operated, I/R and NRIPoC groups (Fig. 1). The I/R group underwent MCAO by occlusion of the right MCA for 90 min, followed by 24 h of cerebral reperfusion. The sham-operated group underwent the same procedure as the I/R rats, but without occlusion of the right MCA. The NRIPoC group underwent the same procedure as the I/R group, but was also subjected to sequential I/R, which involved 3 applications of ischemia for 5 min and reperfusion for 5 min to the right hind limb during MCA recovery reperfusion.

To carry out this procedure, a modified non-invasive blood pressure cuff was strapped to the base of the right hind limb of the rat, and pulse sensors were placed on the area of the dorsalis pedis artery. NRIPoC was induced by increasing the pressure of the cuff until blood flow in the limb was blocked. This was maintained for 5 min and then released for 5 min, beginning the onset of MCA reperfusion. This was carried out 3 times for 10-min cycles. The blood flow of the right hind limb was completely blocked by the cuff as confirmed by the disappearance of the pulse and hypothermia and skin cyanosis in the limb.

### Neurological evaluation

The animals were returned to their cages after the procedures were finished and again allowed free access to food and water. The neurological deficit score (NDS), as previously described by Longa *et al* (46), was measured to assess neurological evaluation at 24 h after reperfusion as follows: 0, no deficit; 1, failure to extend left forepaw fully; 2, circling to the left; 3, falling to the left; and 4, no spontaneous walking with a depressed level of consciousness.

### Measurement of infarct volume

After a reperfusion period of 24 h, the cerebral infarct area was identified by 2,3,5-triphenyltetrazolium chloride (TTC) staining as previously described (47,48). The infarct volume was calculated according to the following formula: V = t × (A1 + A2... + An), where ‘t’ is the brain slice thickness and ‘A’ is the infarct area. The percentage cerebral infarct volume was calculated as follows: (cerebral infarct total volume/whole brain volume) ×100.

### Preparation of cerebral specimens

After a reperfusion period of 24 h, 5 rats in each group were randomly selected and anesthetized with chloral hydrate. Approximately 300–400 ml normal saline was infused via an aortic root catheter until the liver appeared white, followed by 200 ml 4% paraformaldehyde solution that had been cooled to 4°C. The brain was removed, fixed in 4% paraformaldehyde solution for 24 h, and 4-mm-thick coronal sections were then cut from the front of the brain through the optic chiasm. After gradient ethanol dehydration, vitrification with isobutanol and n-butyl alcohol and embedding in paraffin wax, the slices were cut into 5-μm-thick coronal sections on a vibratome, and the sections were dried overnight in an incubator at 37°C for later processing with hematoxylin and eosin (H&E) and Nissl staining, terminal deoxynucleotidyl transferase-mediated dUTP nick end-labelling (TUNEL).

### Assessment of neuronal survival

The coronal brain sections were examined using a standard hematoxylin and eosin (H&E) staining protocol to examine cellular morphology by observing the number and shape of the neurons. The brain sections were stained with toluidine blue for Nissl bodies. Neuronal survival was assessed by observing the Nissl bodies in the neurons. Images of the ischemic penumbras of the 5 rats were captured, and quantitative analyses of the cells were performed using Image-Pro Plus 6.0 software (Media Cybernetics, Inc., Rockville, MD, USA).

### Assessment of apoptosis

Apoptosis was measured by TUNEL assay using an Apoptosis Detection kit (Roche Applied Science, Mannheim, Germany), in accordance with the manufacturer’s instructions. TUNEL-positive neurons were viewed under a light microscope (Leica DM 2000; Leica Microsystems, Wetzlar, Germany) and were identified by bluish-violet-stained nuclei. Five visual fields were randomly selected from the ischemic penumbra of each slice at ×400 magnification. The apoptotic cells were counted in each field, and the average value of the 5 fields was calculated.

### Western blot analysis

After a reperfusion period of 24 h, the brains of the 5 rats in each group selected randomly were rapidly removed under deep anesthesia, and the right ischemic penumbra areas of the parietal cortex were immediately isolated onto ice held at −20°C. The pulverized brain samples weighing 0.25 g were homogenized with 500 μl protein lysis buffer. The homogenates were centrifuged at 12,000 rpm for 5 min at 4°C, and the supernatants were collected for western blot analysis. The protein concentrations were determined by bicinchoninic acid (BCA) protein assays (WellBiz Brands, Inc., Highlands Ranch, CO, USA). Western blot analyses were carried out using standard techniques (49). Briefly, a mixture of homogenate sample containing 30 μg protein and 5× sodium dodecyl sulfate (SDS) loading buffer was boiled at 100°C for 5 min, separated on 10% SDS-polyacrylamide gels, transferred onto polyvinylidene fluoride (PVDF) membranes (Pierce Biotechnology, Inc., Rockford, IL, USA) and blocked for 1 h with 5% non-fat powdered milk in 100 mM Tris-buffered saline containing 0.05% Tween-20 (TBST). The membranes were incubated overnight at 4°C with antibodies against Bcl-2 (Abcam plc., Cambridge, UK), Bax (Cell Signaling Technology, Inc., Beverly, MA, USA), phosphorylated STAT3 (p-STAT3), or β-actin (both Santa Cruz Biotechnology, Inc., Santa Cruz, CA, USA). After 3 washes in TBST, the membranes were incubated with secondary antibody (HRP rabbit anti-goat IgG; Santa Cruz Biotechnology, Inc.) for 1 h at room temperature, followed by 3 washes in TBST. Signals were detected with an Enhanced Chemiluminescence kit (Pierce Biotechnology, Inc.).

### Statistical analysis

Data analyses were performed using SPSS software 13.0 (SPSS Inc., Chicago, IL, USA). NDS values are expressed as the median (range) and were compared using Kruskal-Wallis tests. Other data are presented as the means ± standard deviation (SD). One-way analysis of variance (ANOVA) followed by Student’s t-tests were applied to determine differences between groups. A value of P<0.05 was considered to indicate a statistically significant difference.

## Results

### Effects of NRIPoC on the IRI-induced cerebral infarct volume

**Figure 2:**
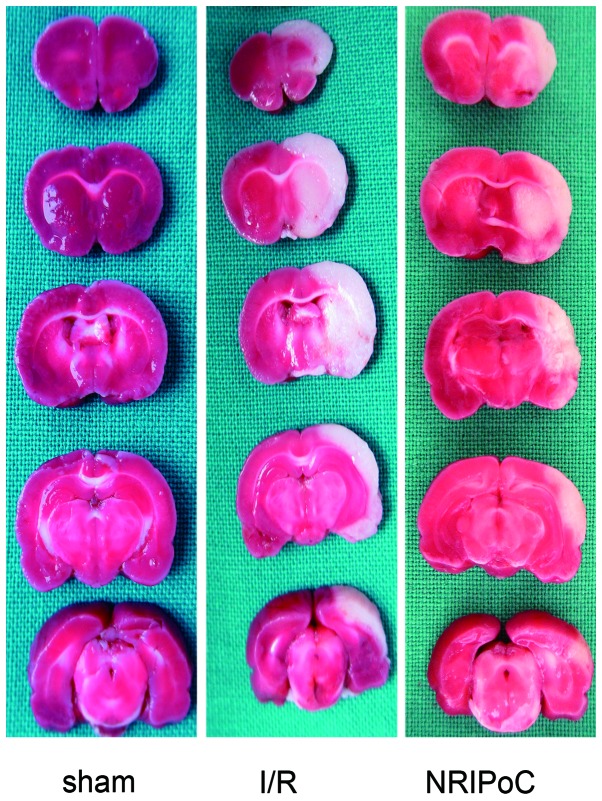
Effects of non-invasive remote ischemic postconditioning (NRIPoC) on the ischemia reperfusion injury (IRI)-induced cerebral infarction volume determined by 2,3,5-triphenyltetrazolium chloride (TTC) staining (n=5); normal brain tissue is red, infarcted brain tissue is white.

The cerebral infarct areas determined by TTC staining are illustrated in Fig. 2. There were no conspicuous cerebral infarcts area in the sham-operated group rats, while the cerebral infarct volumes of the I/R and NRIPoC rats were 28.4±3.7 and 15.2±6.9, respectively. The cerebral infarct volumes were significantly decreased in the NRIPoC group compared with the I/R group (P<0.05).

### Effects of NRIPoC on IRI-induced neuronal loss

**Figure 3:**
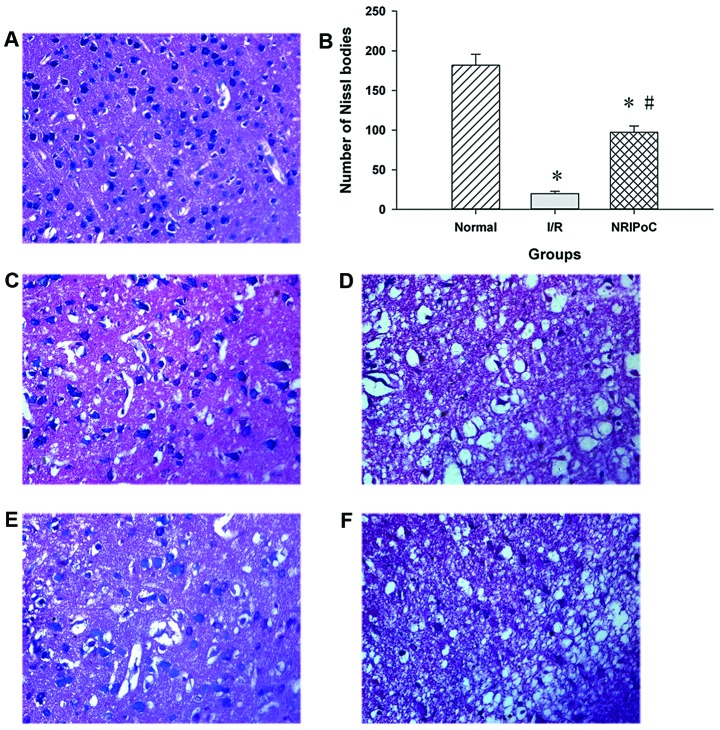
Nissl bodies in the penumbra and core area in the 3 groups at 24 h after reperfusion (n=5). Nissl bodies are shown at ×400 magnification. In the rat cortex, Nissl bodies appear as large granular basophilic bodies in the neuronal cytoplasm and are composed of a rough endoplasmic reticulum. The damaged neurons were identified by the loss of Nissl substance. Data are presented as the means ± standard deviation (SD). ^*^P<0.05 compared with the sham-operated (normal) group; ^#^P<0.05 compared with the ischemia/reperfusion (I/R) group. (A) Cortex in the sham-operated group; (B) statistical histogram of Nissl bodies in the 3 groups; (C) penumbra cortex in the I/R group; (D) ischemic core in the I/R group; (E) penumbra cortex in the non-invasive remote ischemic postconditioning (NRIPoC) group; (F) ischemic core in the NRIPoC group.

Nissl bodies were used as a morphological indicator of neuronal survival. The number of Nissl-stained neurons in the ischemic penumbra areas in the I/R and NRIPoC groups was significantly reduced compared with the sham-operated group (P<0.05); however, this decrease was less significant in the NRIPoC group compared with the marked decrease observed in the I/R group (Fig. 3A–C and E). The Nissl bodies in the ischemic core areas in the I/R and NRIPoC groups were wiped out in vast numbers, and there was no significant difference between the 2 groups (Fig. 3D and F).

### Effects of NRIPoC on IRI-induced neuronal morphological changes

**Figure 4:**
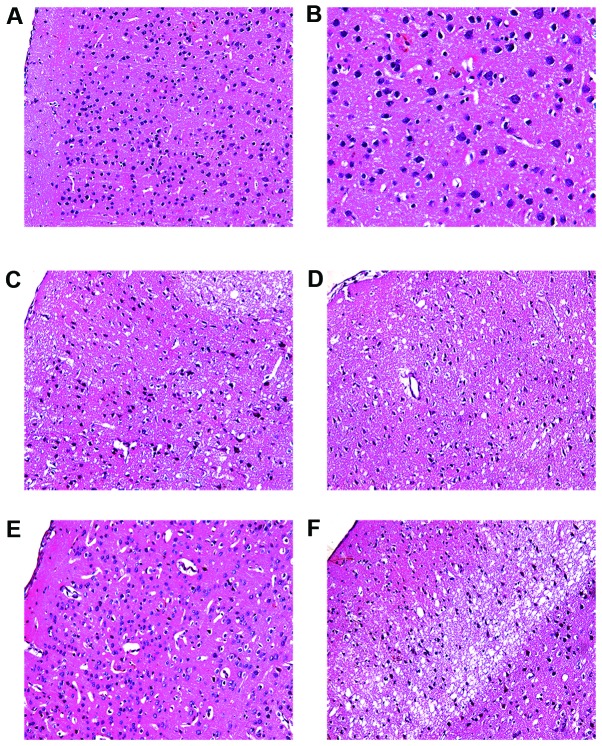
The cortex morphology assessed by hematoxylin and eosin (H&E) staining in the 3 groups at 24 h after reperfusion. All images are ×200 magnification with the exception of (B) (x400). The neuronal morphology of the sham-operated (sham) group cortex was normal. The cellular morphology of the ischemic penumbra tissue in the non-invasive remote ischemic postconditioning (NRIPoC) group was markedly improved compared with the ischemia/reperfusion (I/R) group. There were a few neurons in the ischemic core areas of the I/R and NRIPoC groups. (A and B) Sham-operated group cortex; (C) ischemic penumbra of the I/R group; (D) ischemic core area of the I/R group; (E) ischemic penumbra of the NRIPoC group; (F) ischemic core area of NRIPoC group.

Neuronal morphology was assessed by H&E staining (Fig. 4). The number of neurons in the ischemic penumbra area of the I/R group was reduced. The obvious characteristics of the neurons were: decreased cell size, nuclear pyknosis, interstitial edema, cell disorder and chromatin condensation. Compared with the I/R group, the NRIPoC group showed a marked improvement in the cellular morphology of the ischemic penumbra area, as only a few neurons were observed to have nuclear pyknosis, hyperchromasis and extremely loose organization. There was no obvious difference in cellular morphology in the ischemic core area between the I/R and NRIPoC groups.

### Effects of NRIPoC on IRI-induced neuronal apoptosis

**Figure 5:**
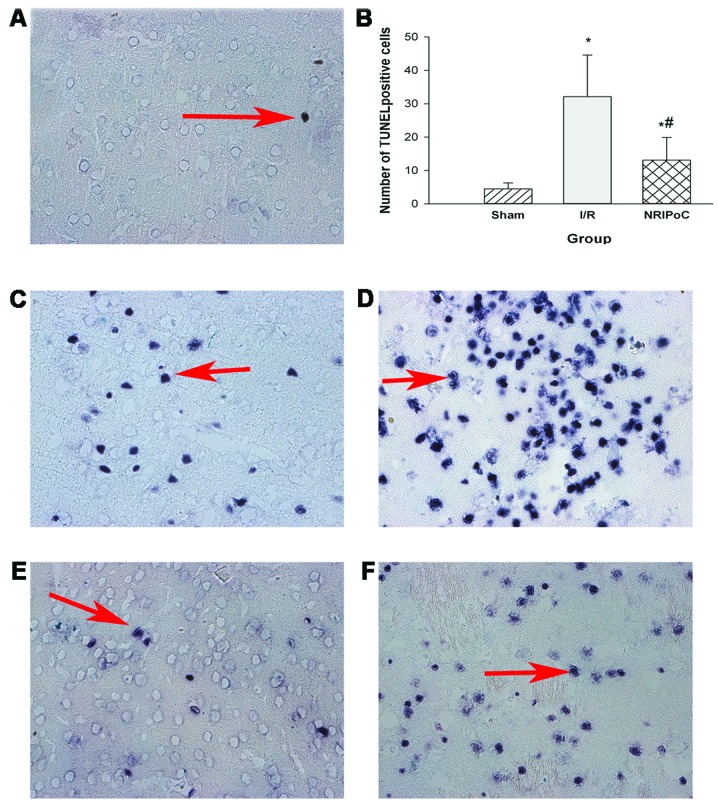
Effects of non-invasive remote ischemic postconditioning (NRIPoC) on ischemia reperfusion injury (IRI)-induced neuronal apoptosis determined by TUNEL staining at 24 h after reperfusion (n=5). The TUNEL-positive cells are shown at ×400 magnification. Arrows indicate TUNEL-positive cells. The features of TUNEL-positive cells observed under a light microscope were as follows: violet granules, uneven nuclei, an irregular shape, nuclear pyknosis and nuclear fragmentation. Data are presented as the means ± standard deviation (SD). ^*^P<0.05 compared with the sham-operated (sham) group; ^#^P<0.05 compared with the I/R group. (A) Sham-operated group cortex; (B) I/R group cortex; (C) ischemic penumbra of the I/R group; (D) ischemic core area of the I/R group; (E) ischemic penumbra of the NRIPoC group; (F) ischemic core area of the NRIPoC group.

Apoptosis was determined by TUNEL staining (Fig. 5), which revealed that there were few apoptotic neurons in the sham-operated group. In the I/R group, there were large numbers of apoptotic neurons in the ischemic penumbra area; however, NRIPoC significantly decreased these numbers (P<0.05). There were numerous apoptotic neurons in the ischemic core areas of both the I/R and NRIPoC groups.

### Effects of NRIPoC on IRI-induced Bcl-2 and Bax expression in the ischemic penumbra area

**Figure 6:**
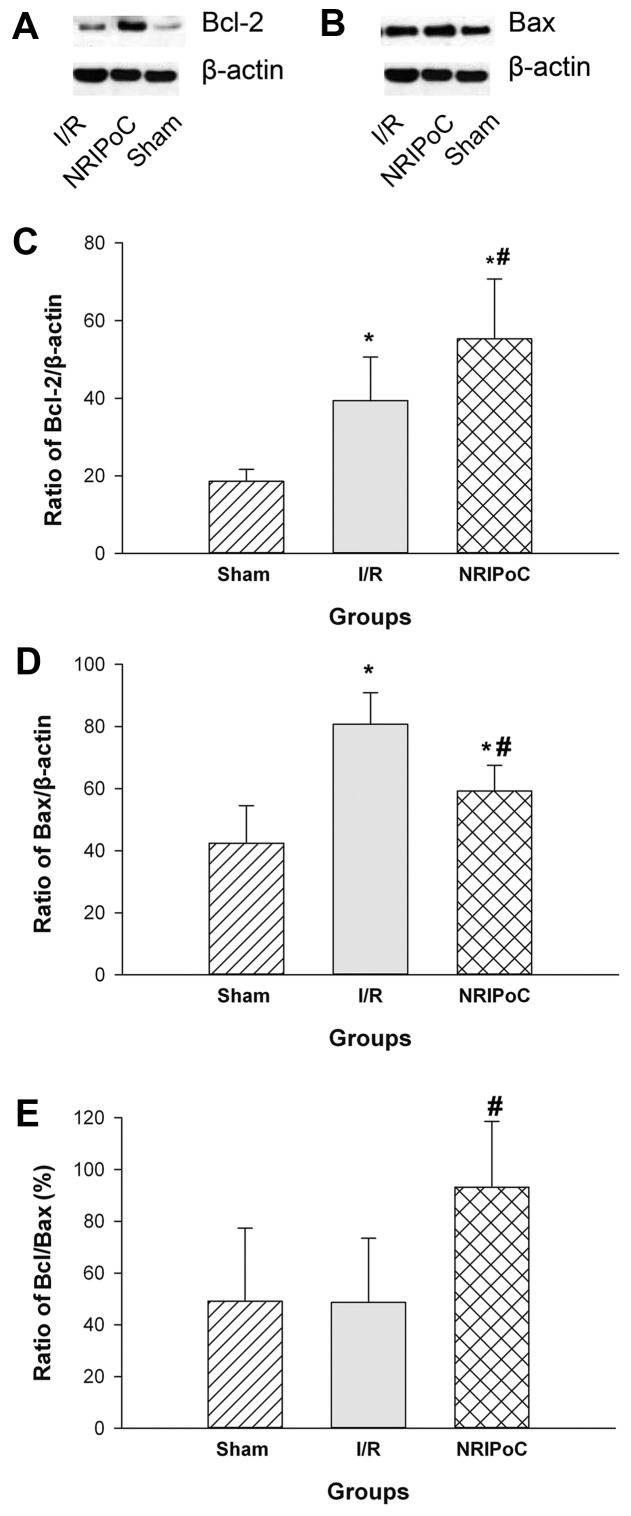
Effects of non-invasive remote ischemic postconditioning (NRIPoC) on ischemia reperfusion injury (IRI)-induced Bcl-2 and Bax protein expression in ischemic penumbra areas 24 h after reperfusion (n=5). (A) Bcl-2 and (B) Bax protein expression determined by western blot analysis. (C) Ratio of Bcl-2/β-actin; (D) ratio of Bax/ β-actin; (E) ratio of Bcl-2/Bax. Data are presented as the means ± standard deviation (SD). ^*^P<0.05 compared with the sham-operated (sham) group; ^#^P<0.05 compared with the ischemia/reperfusion (I/R) group.

Bcl-2 and Bax protein levels were significantly increased in the I/R and NRIPoC groups compared with the sham-operated group (P<0.05) (Fig. 6). NRIPoC significantly increased Bcl-2 protein expression and decreased Bax protein expression following IRI (P<0.05). There was no difference in the Bcl-2/Bax ratio between the I/R and sham-operated groups; however, this ratio was significantly increased in the NRIPoC group compared with the I/R group (P<0.05).

### Effects of NRIPoC on p-STAT3 protein expression in the ischemic penumbra area

**Figure 7:**
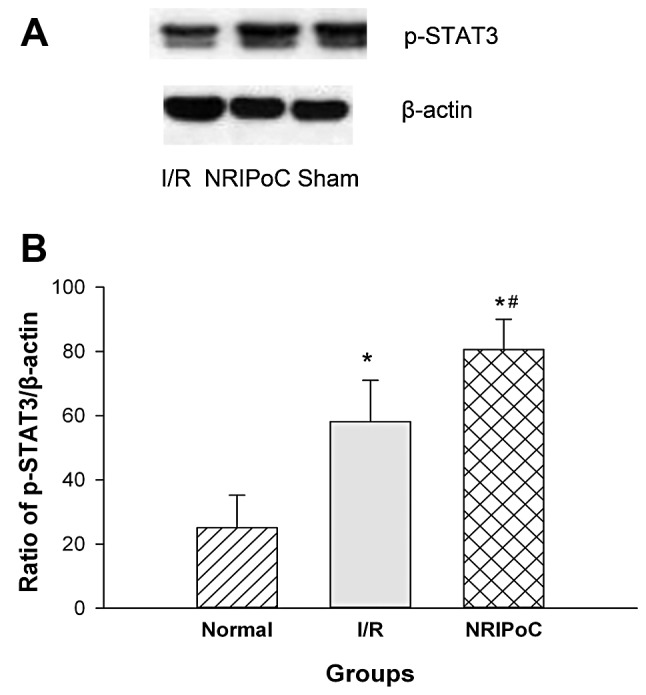
Effects of non-invasive remote ischemic postconditioning (NRIPoC) on ischemia reperfusion injury (IRI)-induced p-STAT3 protein expression in ischemic penumbra areas 24 h after reperfusion (n=5). (A) p-STAT3 protein expression determined by western blot analysis. (B) Ratio of p-STAT3-β-actin. Data are presented as the means ± standard deviation (SD) (n=5). ^*^P<0.05 compared with the sham-operated (normal) group; ^#^P<0.05 compared with the ischemia/reperfusion (I/R) group.

The expression of p-STAT3 protein in the ischemic penumbra area was significantly increased following IRI (P<0.05) (Fig. 7). NRIPoC significantly increased p-STAT3 protein expression in the ischemic penumbra area compared with the I/R group (P<0.05).

### Effects of NRIPoC on I/R-induced NF-κB and TNF-α expression in the ischemic penumbra area

**Figure 8:**
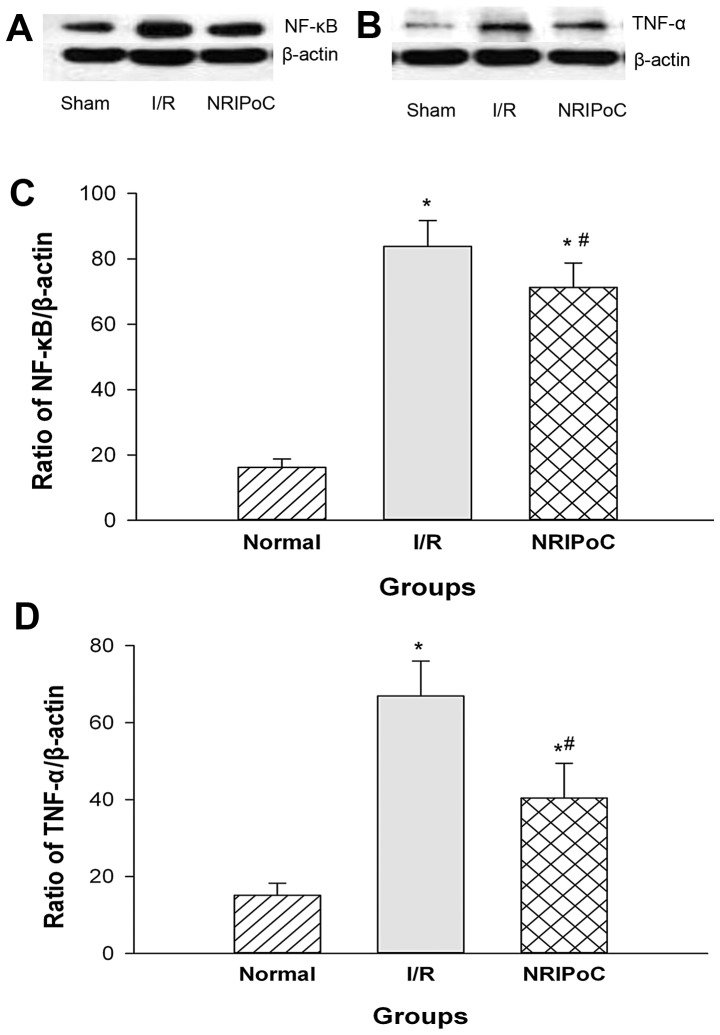
Effects of non-invasive remote ischemic postconditioning (NRIPoC) on ischemia reperfusion injury (IRI)-induced nuclear factor-κB (NF-κB) and tumor necrosis factor-α (TNF-α) protein expression determined by western blot analysis in the ischemic penumbra 24 h after reperfusion (n=5). (A) NF-κB protein expression; (B) TNF-α protein expression. (C) Ratio of NF-κB/β-actin; (D) Ratio of TNF-α/ β-actin. Data are presented as the means ± standard deviation (SD). ^*^P<0.05 compared with the sham-operated (normal) group; ^#^P<0.05 compared with the ischemia/reperfusion (I/R) group.

The NF-κB and TNF-α protein expression levels in the ischemic penumbra area were significantly increased following IRI (P<0.05) (Fig. 8). NRIPoC significantly decreased NF-κB and TNF-α expression compared with the I/R group (P<0.05); however, the levels did not return to those of the sham-operated group (P<0.05).

### Effects of NRIPoC on neurological function

Neurological function in the sham-operated group was normal (NDS =0) (Table I). The median NDS of the NRIPoC group was 1 score lower on the scale than that in the I/R group, but there was no statistically significant difference between the 2 groups (P>0.05), and the median NDS in the NRIPoC group was still higher than that of the sham-operated group (P<0.01).

## Discussion

In this study, we used an *in vivo* model of MCAO-induced focal ischemia, which has the advantage of being simple and reliable without requiring a craniectomy. MCAO has been widely used to study the effects and mechanisms of pre- and postconditioning on focal cerebral ischemia. Although there have been various ischemic penumbra definitions following focal cerebral ischemia put forward over the 30 years since ischemic penumbra was first introduced by Astrup *et al* (50) in 1981, salvaging the ischemic penumbra is the current primary therapeutic target in acute stroke (51). Following cerebral ischemia reperfusion, many neurons in the ischemic penumbra or peri-infarct zone may undergo apoptosis after several hours or days. This renders the time window of therapeutic opportunity for salvaging those cells of the ischemic penumbra.

We hypothesized that the neuroprotective effects of NRIPoC may be mediated through its effects on the ischemic penumbra. The results from the present study demonstrate that NRIPoC significantly increased the number of surviving neurons and decreased neuronal apoptosis in the ischemic penumbra following IRI in a rat model of focal cerebral ischemia induced by 90 min of MCAO. Conversely, there was no difference in neuronal survival in the ischemic core between the I/R and NRIPoC groups. The finding that NRIPoC significantly decreased neuronal apoptosis in the penumbra are is consistent with the results of a previous study demonstrating that RIPoC significantly attenuated neuronal apoptosis associated with global cerebral ischemia in the hippocampal CA1 region (19). TTC is a light-sensitive compound that reacts with lactate dehydrogenase (LDH), which stains normal brain tissue red, while ischemic brain tissue remains white. In the present study, TTC staining revealed that NRIPoC significantly reduced the cerebral infarct size, suggesting that NRIPoC increased cell viability in the cerebral penumbra area. This observation is in accordance with the results of previous studies by Ren *et al* (13,17).

In addition to necrosis, there is overwhelming evidence suggesting that apoptosis significantly contributes to cell death following cerebral IRI (27,52,53). Preventing apoptosis in the penumbra area may reduce neuronal loss and limit cerebral IRI. Ischemia pre- and postconditioning has been shown to decrease neuronal apoptosis and attenuate IRI in brain tissue (6,54–57). Similarly, NRIPoC significantly decreased neuronal apoptosis and reduced the cerebral infarct volume after focal cerebral ischemia in the present study. These results suggest that anti-apoptotic signaling may be an important mechanism responsible for the neuroprotective effects of NRIPoC.

Bcl-2 family members are key regulators of apoptosis and modulate mitochondrial membrane permeability. Following cell ischemia or hypoxia, the expression of mitochondrial membrane proteins is increased, and the apoptotic protein, Bax, which translocates from the cytoplasm into the mitochondria, promotes the release of cytochrome *c*, while the anti-apoptotic protein, Bcl-2, located in the mitochondrial wall, inhibits the release of cytochrome *c* (58–60). Cytochrome *c* initiates the process of apoptosis by activating the caspase cascade (59). Interactions between the pro- and anti-apoptotic Bcl-2 family proteins on the outer mitochondrial membrane play a key role in cell survival. Therefore, the Bcl-2/Bax protein ratio may determine the level of cell apoptosis or survival following apoptotic injury (61). In the present study, NRIPoC significantly decreased both Bax and Bcl-2 protein expression and increased the Bcl-2/Bax ratio. These results suggest that the anti-apoptotic effects of NRIPoC may be closely associated with changes in Bcl-2 and Bax expression. The mechanisms through which NRIPoC modulates Bcl-2 and Bax expression following cerebral IRI *in vivo* are currently unknown. The JAK/STAT signaling pathway transduces stress-activating extracellular chemical signals into cellular responses for a number of pathophysiological processes, such as immunity, inflammation and apoptosis (62). p-STAT1 and p-STAT3 expression have been shown to be increased following focal cerebral ischemia, and the activation of STAT1 may lead to ischemic neuronal necrosis (42,43,63); however, the effects of activated STAT3 following focal cerebral ischemia have not yet been elucidated. The function of activated STAT3 is controversial; some studies have associated it with survival (43,64), while others have related it to cell death (65).

There is no existing evidence that STAT3 is involved in neuronal apoptosis by the regulation of Bcl-2 and Bax expression following cerebral ischemia. Previous studies have confirmed that STAT3 alterations affect Bcl-2 and Bax protein expression (decreased Bcl-2 and increased Bax) and induce inflammation and apoptosis in many types of tumor cells (66–68). Lee *et al* (69) demonstrated that co-activated NF-κB and STAT3 modulate Bax/Bcl-xL expression and promote cell survival in head and neck squamous cell carcinoma.

In mycosis fungoides tumor cells, some apoptosis-related genes, such as Bcl-2 and Bax, have been identified as STAT3 target genes (66). In primary cortical neurons and murine models of stroke, the activation of the Jak2/Stat3 pathway by secretoneurin has been found to exert neuroprotective effects and induce neuronal plasticity after hypoxia and ischemic insult (70). Using a mouse model of transient focal cerebral ischemia, Jung *et al* (71) demonstrated that interleukin-6 (IL-6) exerted protective effects against cerebral ischemic injury through IL-6R-mediated STAT3 activation and antioxidative signaling. Similar to previous study by Suzuki *et al* (43), we found that p-STAT3 expression was increased following focal cerebral ischemia. Furthermore, NRIPoC induced a more pronounced increase in p-STAT3 expression in the ischemic penumbra area. This, coupled with the Bcl-2/Bax ratio was consistent with the decrease in the number of apoptotic neurons and the attenuation of cerebral I/R-induced brain injury. This result provides an experimental basis for the clinical application of NRIPoC for reducing the cerebral infarct volume following stroke through the rescue of the remaining surviving neurons in the penumbra area. These results indicate that p-STAT3 may be an endogenous anti-injury factor, and that the JAK2-STAT3 signaling pathway may mediate the neuroprotective effects of RIPoC though the regulation of Bcl-2/Bax expression in rat focal cerebral ischemia. This assumption requires further confirmation by the use of specific inhibitors of the JAK2-STAT3 signaling pathway in order to clarify the effects of STAT3.

The pathogenesis of cerebral IRI involves complex pathophysiological events, such as the excessive production of reactive oxygen species, the excessive activation of glutamate receptors, the overload of intracellular calcium and inflammation (26,27,72). Early inflammatory mediators following cerebral IR are the basis of ischemic injury that then transforms to inflammatory injury. Accumulating evidence suggests that the neuroinflammatory processes occurring after cerebral ischemia involve various pathways and molecules (26). Of these, TNF-α is responsible for the degree of inflammation. NF-κB is a critical transcription factor for the maximal expression of several cytokines that are involved in inflammation (73). Whereas TNF-α and IL-1β appear to exacerbate cerebral injury, transforming growth factor-β and IL-10 may exert neuroprotective effects during cerebral ischemia reperfusion (26). Activated NF-κB upregulates the transcription of TNF-α and IL-1β. An increase in TNF-α activates NF-κB, and IL-10 inhibits the activation of NF-κB mediated by endotoxins. The synthesis of inflammatory mediators is greatly increased by the overexpression of TNF-α following I/R, which results in an imbalance between inflammatory and anti-inflammatory factors and the amplification of the inflammatory cascade. The overexpression of TNF-α may aggravate brain damage following cerebral I/R, which is though to be mediated by the transcriptional regulation of inflammatory factors by NF-κB (26,74). The present study demonstrated that TNF-α and NF-κB protein expression in the ischemic penumbra area was significantly increased following I/R, and this increase was reduced by NRIPoC, which suggests that the neuroprotective effects of NRIPoC may correlate with the increased inflammatory response in the ischemic penumbra area following IRI. The binding of TNF-α to its receptor, TNF-RI (p55TNFR), induces the caspase cascade, which promotes apoptosis. Several alternative mechanisms of TNF-α-induced apoptosis have also been described (75). The downregulation of TNF-α by NRIPoC in the ischemic penumbra area may be one of the reasons why NRIPoC reduced neuronal apoptosis following cerebral I/R. Further studies are required to examine the role of STAT3 signaling in regulating TNF-α and NF-κB expression induced by cerebral I/R, as well as the neuroprotective effects of NRIPoC.

In conclusion, the present study demonstrates that NRIPoC attenuates cerebral IRI in an *in vivo* rat model through the alleviation of inflammation, regulation of the STAT3 signaling pathway and Bcl-2 and Bax expression levels, and that these changes are possibly due to the reduction of apoptosis.

## Figures and Tables

**Table I:** Neurological deficit score of the rats in this study.

Group	Neurological deficit score					Median (minimum-maximum)

	0	1	2	3	4	
Sham	15	0	0	0	0	0
I/R	0	0	5	10	0	3 (2–3)
NRIPoC	0	3	6	6	0	2 (1–3)

Sham, sham-operated; I/R, ischemia/reperfusion; NRIPoC, non-invasive remote ischemic postconditioning.
